# Study on the macro–micro evolution of compaction characteristics of coal gangue with different grain sizes under side-limit compression conditions

**DOI:** 10.1038/s41598-024-54804-4

**Published:** 2024-02-28

**Authors:** Zhenzhi Liu, Ping Liu, Zhen Lu, Jian Li, Chang Luo

**Affiliations:** 1 College of Mining, Guizhou University, Guiyang, 550025 Guizhou China; 2 School of Civil Engineering and Architecture, Guizhou Minzu University, Guiyang, 550025 Guizhou China; 3 Guizhou Jinsha Longfeng Coal Co, Bijie, 551800 Guizhou China; 4 Guizhou Woze Engineering Management Consulting Co, Qianxinan, 562400 Guizhou China

**Keywords:** Gangue, Numerical simulation, Compression change, Mechanical properties, Environmental sciences, Natural hazards, Engineering, Materials science

## Abstract

Currently the main method of mine solid waste disposal and utilization is to apply gangue to underground filling. For the grading characteristics of coal gangue in the hollow zone in underground filling, the state of pressure and the mechanical properties of coal gangue filling materials under different particle sizes. In this paper, through laboratory tests and numerical simulations, the bearing characteristics and deformation damage of gangue are deeply investigated and analyzed from both macroscopic and microscopic perspectives. The results of the study show that when the axial pressure reaches a certain threshold, the strain increases accordingly with the increase in grain size. Under the axial pressure condition of 15MPa, the bearing capacity of gangue with different grain sizes under the lateral pressure of steel cylinder showed obvious positive correlation. In the software PFC3D simulation, as the particle size of gangue particles increases, it has a significant effect on the natural stacking porosity of the specimen, and gangue particles are more prone to force chain instability and failure. Mixed particle size gangue can significantly reduce the natural accumulation porosity of the specimen, thus improving its overall stability. Significant displacement triangles existed in the top and lateral directions of the B1 (0–5 mm), B2 (5–10 mm), and B5 (0–20 mm) specimens under the same circumferential pressure conditions. The results of the study are of great significance for further selection of reasonable gangue gradation and determination of its mechanical properties.

## Introduction

With the long-term sustained development of coal resources, the continuous underground coal mining activities have caused surface subsidence and accumulation of coal gangue waste, which has seriously affected coal mining and caused serious pollution to the living environment in mining areas^1–4^. Fill mining, as a green mining technology, utilises coal gangue underground for filling in order to reduce problems such as difficulty in accumulating coal gangue on the surface and pollution of the environment, to improve the recovery rate of coal resources and to control surface subsidence^5–7^. In the process of filling and mining, the gangue pressure-bearing deformation characteristics of the quality and effect of filling and mining has a decisive impact, directly affecting the surface subsidence and roof control and other engineering problems, need to carry out in-depth research on its characteristics to ensure the safety of filling and mining operations^8–11^. In the current research, a large number of studies have been carried out on the compressive macroscopic deformation characteristics of crushed gangue, and its research mechanical behavior mainly includes expansion, stress–strain, densification and compression damage, etc. At the same time, the influence of factors such as material ratio, particle size distribution and loading conditions has also been thoroughly explored^12–16^. Therefore, the deformation characteristics of coal gangue with different particle sizes under confined compression conditions are studied by obtaining the pressure-bearing mechanical parameters of coal gangue through laboratory experiments, and the microscopic evolution mechanism of filling materials is further revealed, which provides an important basis for the selection of filling mining materials and is of great significance for improving the safety of filling mining^17–19^.

At present, domestic and foreign scholars have carried out in-depth discussion and a lot of research on the pressure deformation characteristics of coal gangue^20,21^. With the increase of mining depth, the stress of the original rock also increases, the filling material needs better compression resistance, so it is especially important to improve the denseness of the gangue filling material^22^. Huang et al.^23^ used PFC3 D software and selected Clump model to generate irregular geometric gangue particles. Under the condition of confined compression, the lateral pressure of broken gangue is proportional to the axial pressure. With the increase of confining pressure, the bearing capacity of gangue will be enhanced accordingly. Banerjee et al.^24^. conducted conventional triaxial tests on the engineering mechanical properties of cohesive soils and related numerical simulations of 3D granular flow, comparing the stress–strain relationship curves of 3D granular body specimens with the indoor triaxial at different confining pressures, respectively, and basically fitting and loading curves of the indoor tests and numerical simulations. Yu et al.^25^ explored the gangue damage characteristics through the side-limit compression test, and the research results showed that the contact force between gangue and gangue gradually changed from isotropic to anisotropic, and the failure mode under the contact force of gangue was mainly through the failure of single-pass cracks and the failure of multi-short cracks. Li et al.^19^ used PFC3D model to simulate and analyse the compression deformation characteristics of coal gangue under different particle sizes, and the research results show that the compression deformation has a significant regularity, and the distribution of the particle clusters also presents certain characteristics, at the same time, the research found that the greater the hardness of the gangue filling material, the better the effect of its filling, and further emphasizes the importance of the particle size grading of the crushed gangue particles. Wu et al.^26^ performed the compression tests on red sandstone with different grain sizes through laboratory tests and numerical simulations, and carried out comparative analyses from both macroscopic and microscopic perspectives, resulting in a stable structure that evolves from polygons to triangles between particles. Li et al.^27^ used CT scanning and three-dimensional reconstruction technology to obtain a real gangue three-dimensional model, through the establishment of a triaxial compression numerical model to analyse the macroscopic distortion and microstructure evolution of different shapes of gangue in triaxial compression. Gangue material in the process of bearing pressure, subject to extrusion movement occurs crushing deformation, particles of the deformable space affects the mechanical properties of the gangue, crushed gangue bearing characteristics there is an obvious size effect, indoor testing to obtain the bearing capacity of the academic parameters is difficult to represent the actual mechanical properties on the engineering scale^28–32^.

In summary, this paper studies the stress and strain of coal gangue in the compression process through the confined compression test of coal gangue with different particle sizes in the laboratory. With the help of PFC3D particle flow numerical simulation software, in-depth investigation of different particle size gangue natural accumulation porosity, axial deformation, displacement change, force chain evolution and other factors change on the compression and deformation characteristics, as well as the analysis of the macromechanics behaviour of the gangue and the characteristics of the microscopic movement. Explore the deformation characteristics of gangue filling materials in the compaction process, and then reveal the compressive deformation mechanism of gangue filling body from the microscopic level, optimise the underground filling of coal gangue, and further improve the control of rock movement and surface settlement in the process of filling and mining to provide theoretical basis.

## Tests

### Test materials

#### Determination of maximum particle size

According to literature^33^, the relationship between the diameter (d) of a coarse-grained soil specimen and its grain size should be satisfied as *d*_*max*_ ≤ D**/**5. Where, *d*_*max*_ denotes the maximum grain size of the specimen and D denotes the diameter of the coarse-grained soil specimen. In this test, the inner diameter of the steel cylinder of the test device is 100 mm, according to the above relationship, the maximum size of the gangue screened should be 20 mm.

#### Test material preparation

**Figure 1:**
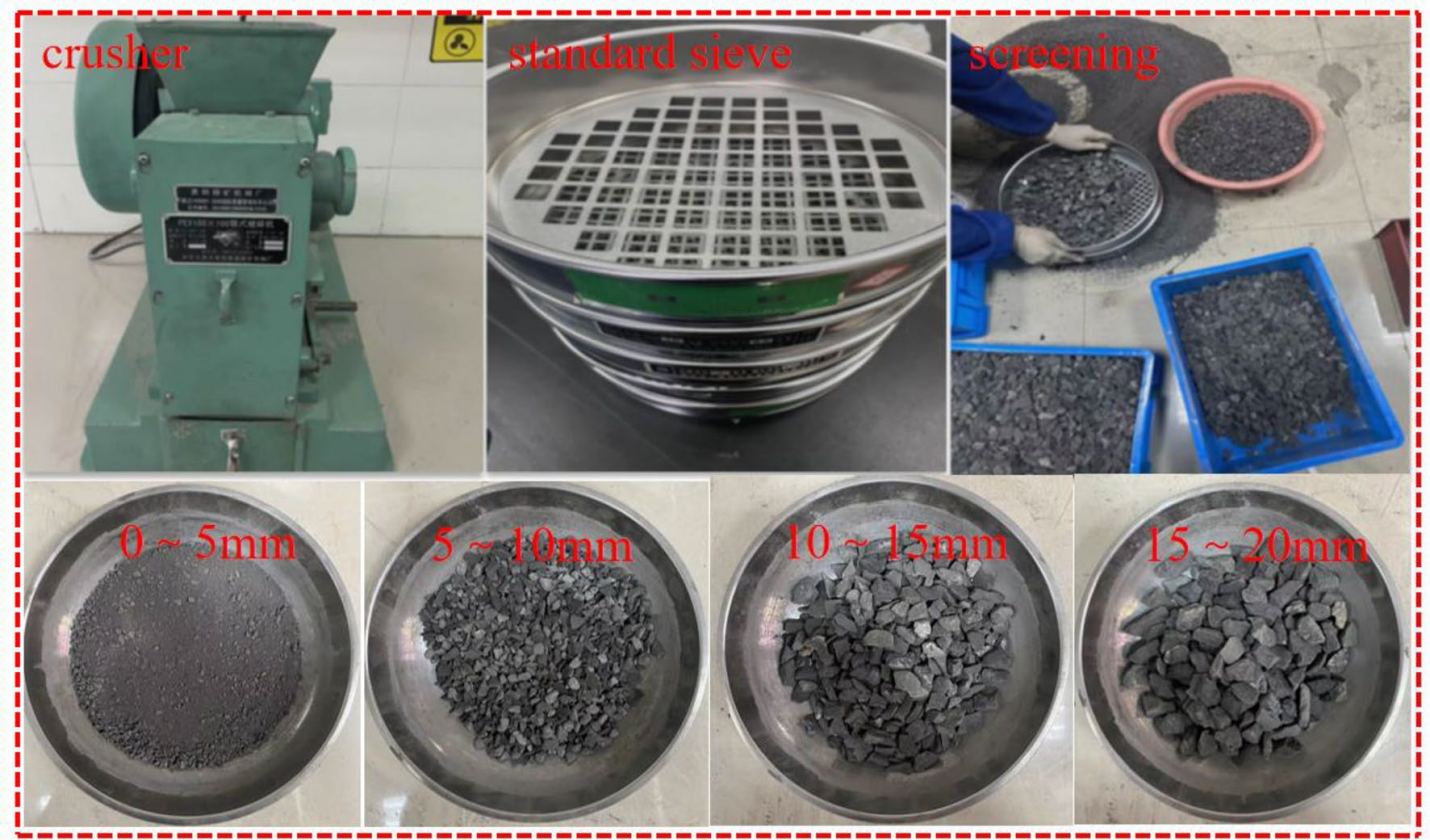
Crushing and screening of gangue in goaf.

The gangue of the mining area used in this test comes from the direct top rock of the Chongyuan coal mine, which is mainly grey-black grey sandstone. The larger gangue is selected for crushing and then screened through national standard square hole screens of different aperture sizes. The nominal diameters of the square hole sieve are 5 mm, 10 mm, 15 mm and 20 mm. After crushing and screening, the gangue of the mining area with particle size distribution of 0 mm to 5 mm, 5 mm to 10 mm, 10 mm to 15 mm, 15 mm to 20 mm and more than 20 mm is obtained. Because the maximum size of the gangue screened in this test is 20 mm, so the gangue larger than 20 mm is crushed for the second time and then continue to be screened, the gangue crushing and screening process in the empty area is shown in Fig. 1.

After crushing and screening, the percentage of (0–20 mm) mixed particle size is shown in Table 1.

**Table 1:** (0–20 mm) Mixed particle size percentage statistics.

particle size distribution	0–5 mm	5–10 mm	10–15 mm	15–20 mm
Particle mass (kg)	34.966	28.520	45.199	16.682
Percentage of particle size	27.89%	22.75%	36.05%	13.31%

### Tests on the basic properties of gangue materials

**Figure 2:**
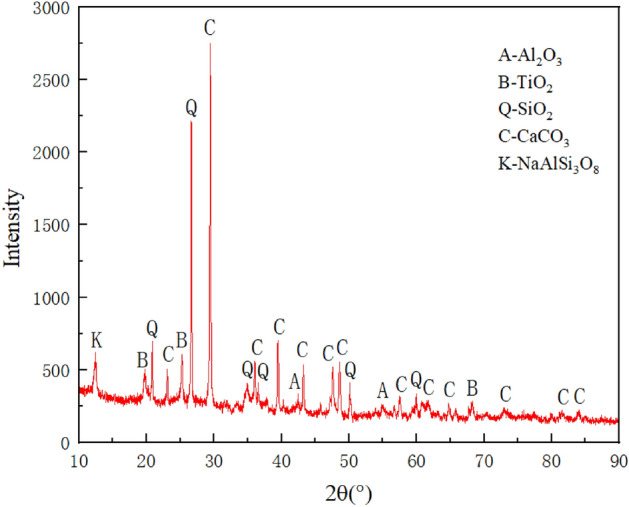
XRD spectra of gangue-filled materials.

As shown in Fig. 2, the composition of gangue was analysed by RXD spectroscopy in this experiment. The experimental results show that the main components of gangue include oxides of silicon, aluminium, calcium and titanium, with calcite having the largest percentage. In addition to this, small amounts of sodium feldspar were found to be present. The chemical composition of gangue mainly consists of Al_2_O_3_, TiO_2_, SiO_2_, CaCO_3_ and NaAlSi_3_O_8_. Due to the high stability and hardness of SiO_2_, the gangue exhibits high hardness and resistance to deformation. A-Al_2_O_3_ (aluminum trioxide), B-TiO_2_ (titanium dioxide), Q-SiO_2_ (silicon dioxide), C-CaCO_3_ (calcium carbonate), K-NaAlSi_3_O_8_ (sodium feldspar).

### Test set-up and programme

When the gangue fills the goaf, the gangue pressure in the goaf gradually tends to be stable, and the maximum pressure comes from the original rock stress. Therefore, combined with the actual background of the coal mine, this test programme was designed to conduct compression tests at three stress levels, namely 5 MPa (200 m filling), 10 MPa (400 m filling) and 15 MPa (600 m filling). At the same time, the stress–strain characteristics of four single particle sizes of 0–5 mm, 5–10 mm, 10–15 mm, 15–20 mm and mixed particle sizes of equal mass of 0–20 mm at three stress levels are investigated. The steel cylinder was filled to a height of 150 mm and the total mass of the mixed grains was 1.82 kg based on the packing density obtained from the results of the previous tests.

In the actual situation, the gangue of the extraction zone will be continuously compressed and deformed after being subjected to the axial pressure given by the basic top, and the axial compression will lead to the expansion and deformation in the ring direction. The expansion of the gangue in the mining area will produce a certain lateral pressure on the gangue retaining wall in the mining area and the filling body next to the roadway, and a certain degree of lateral pressure restricts the lateral movement of the gangue retaining wall, which is conducive to maintaining the integrity of the gangue filling body, but if the lateral pressure is too large, it will cause the instability of the gangue retaining wall. Therefore, it is necessary to carry out the determination of compression lower lateral pressure on the gangue of the extraction zone.

**Figure 3:**
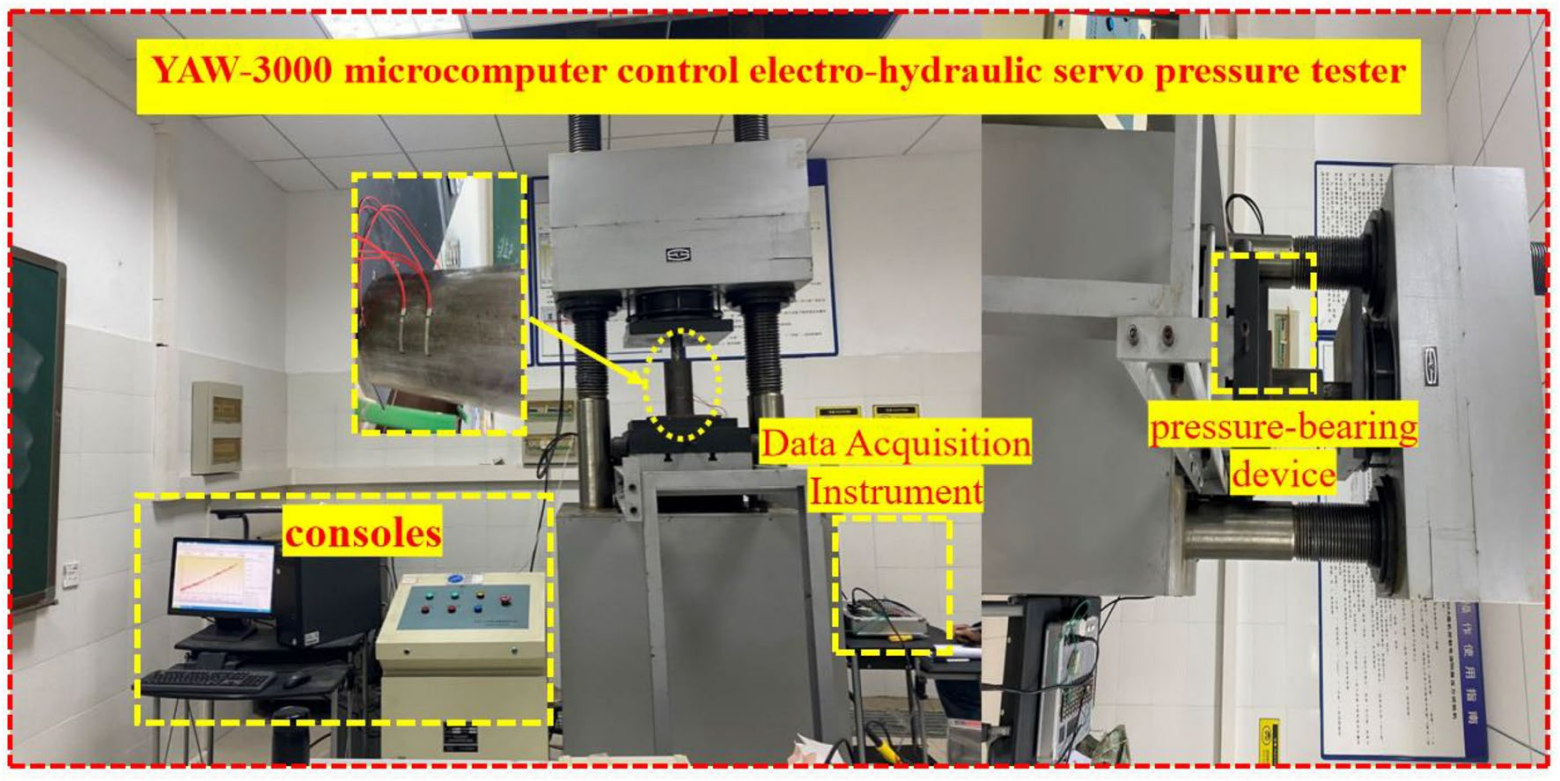
Test system.

The lateral pressure determination test is carried out by YAW-3000 microcomputer-controlled electro-hydraulic servo pressure testing machine, and the axial stress and displacement are collected and recorded by the testing machine. At the same time, two sets of resistance strain gauges were attached at the 1/2 and lower 1/3 of the steel cylinder, and the strain values were recorded with a data acquisition instrument, and finally the average of the four measured strain values was taken. The test system is shown in Fig. 3.

### Calculation of lateral pressure

According to the Lame 's solution formula of cylinder compression in elastic mechanics^33^.

1 $$ {\sigma_\rho } = \frac{{{q_1}{a^2} - {q_2}{b^2}}}{{{b^2} - {a^2}}} - \frac{{\left( {{q_1} - {q_2}} \right){a^2}{b^2}}}{{\left( {{b^2} - {a^2}} \right){\rho^2}}} $$

2 $$ {\sigma_\varphi } = \frac{{{q_1}{a^2} - {q_2}{b^2}}}{{{b^2} - {a^2}}} + \frac{{\left( {{q_1} - {q_2}} \right){a^2}{b^2}}}{{\left( {{b^2} - {a^2}} \right){\rho^2}}} $$

In the formula: ${\sigma_\rho }$ Cylinder axial stress; ${\sigma_\varphi }$ Cylinder transverse stress; *q*_1_ Pressure on the inner wall of the cylinder; *q*_*2*_ Pressure on the outer wall of the cylinder; *a* Cylinder inner wall radius; *b* Cylinder outer wall radius.

In this experiment, the outer wall of the steel cylinder is not stressed, that is, *q*_*2*_ = 0, and the steel cylinder is only affected by *q*_*1*_. Therefore, the axial stress and transverse stress of the steel cylinder at this time are:

3 $$ {\sigma_\rho } = \frac{{{a^2}{q_1}}}{{{b^2} - {a^2}}}\left( {1 - \frac{{b^2}}{{\rho^2}}} \right) $$

4 $$ {\sigma_\varphi } = \frac{{{a^2}{q_1}}}{{{b^2} - {a^2}}}\left( {1 + \frac{{b^2}}{{\rho^2}}} \right) $$

When *ρ* = *b,* Equation (4) can be written as:

5 $$ {\sigma_\varphi } = \frac{{2{a^2}{q_1}}}{{{b^2} - {a^2}}} $$

At the same time, the axial stress of the outer wall of the steel cylinder ${\sigma_\rho }$ = 0, so the force of the outer wall of the steel cylinder is only ${\sigma_\varphi }$.

Because the compression deformation of steel cylinder is a plane strain problem, the Hooke 's law in polar coordinates is:

6 $$ {\varepsilon_\rho } = \frac{{1 - {\upsilon^2}}}{E}\left( {{\sigma_\rho } - \frac{\upsilon }{1 - \mu }{\sigma_\varphi }} \right) $$

7 $$ {\varepsilon_\varphi } = \frac{{1 - {\upsilon^2}}}{E}\left( {{\sigma_\varphi } - \frac{v}{1 - \upsilon }{\sigma_\rho }} \right) $$

In the formula: *υ* Poisson 's ratio of steel cylinder, take 0.25, *E* the elastic modulus of the steel cylinder is 205 GPa.

At this time, the transverse strain of the steel cylinder can be measured by the strain acquisition instrument. The elastic modulus and Poisson 's ratio of the cylinder are known. The lateral pressure of the steel cylinder during the gangue compression test can be obtained by combining Formula (5) and Formula (6):

8 $$ {q_1} = \frac{{E{\varepsilon_\varphi }\left( {{b^2} - {a^2}} \right)}}{{2{a^2}\left( {1 - {\upsilon^2}} \right)}} $$

### Test results and analyses

#### Gangue filling and compacting characteristics analysis

**Figure 4:**
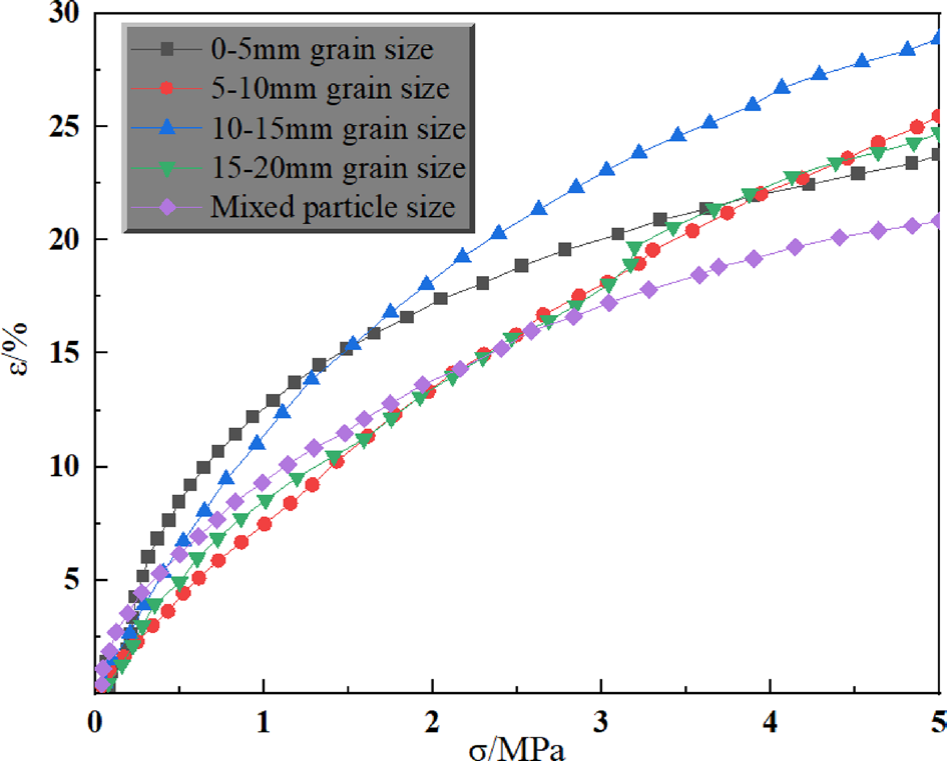
Stress–strain curves of different grain sizes under 5 MPa axial pressure.

**Figure 5:**
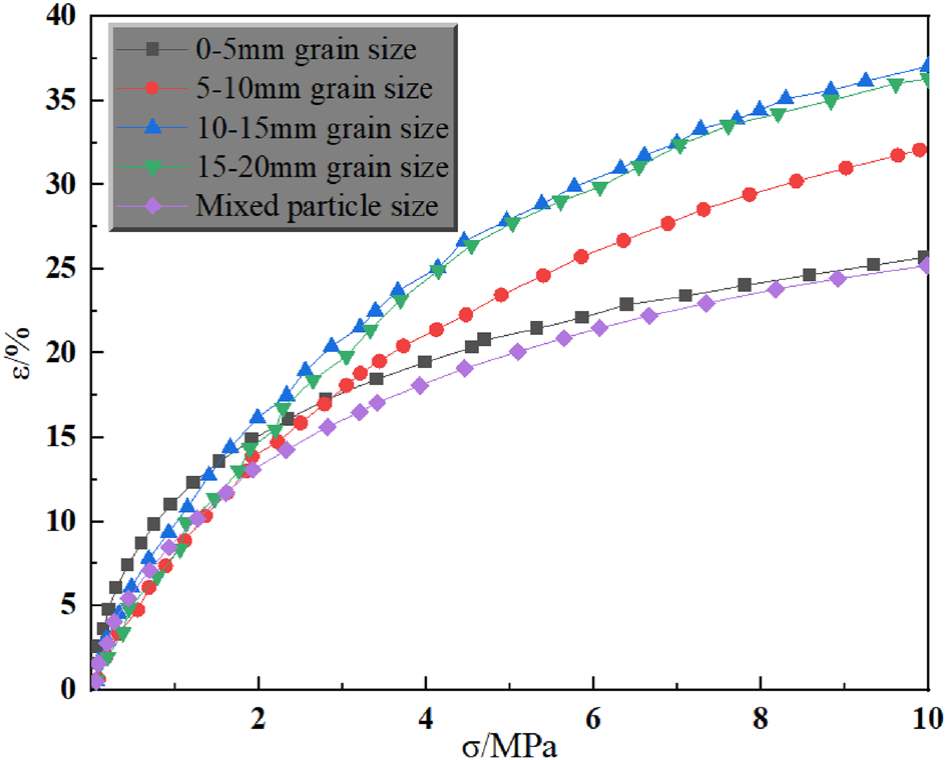
Stress–strain curves of different grain sizes under 10 MPa axial pressure.

**Figure 6:**
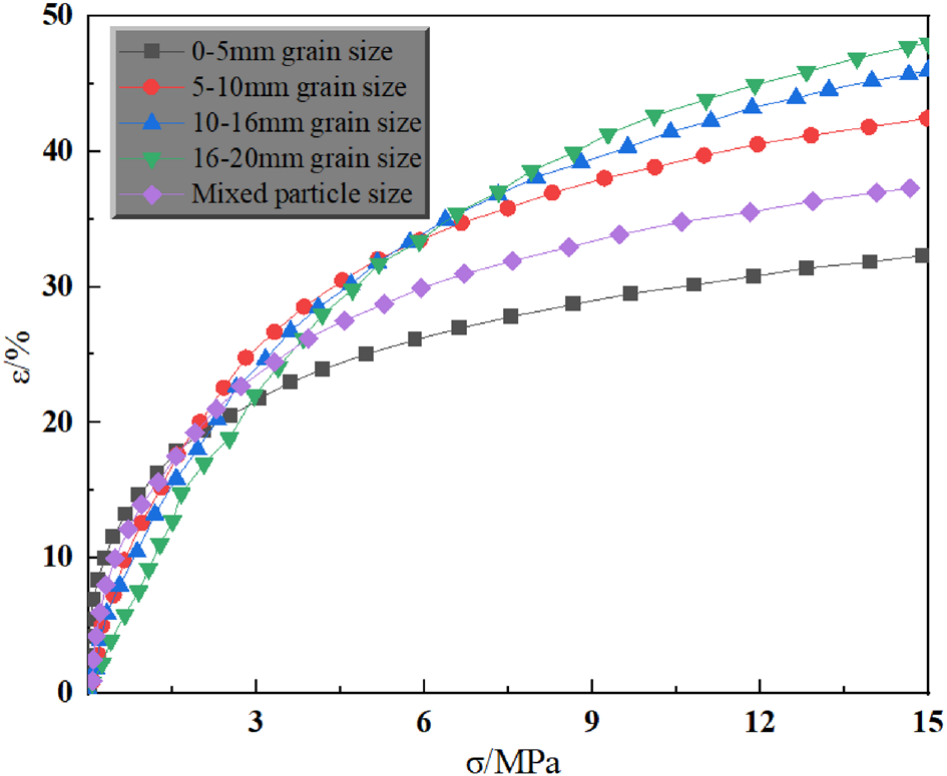
Stress–strain curves of different grain sizes under 15 MPa axial pressure.

In the compression test of gangue in the mining area, the stress–strain curve relationship during compression can reflect the relationship between axial pressure and compression rate, and can also show the residual crushing coefficient of crushed gangue to a certain extent. The compression tests were carried out at three axial stress levels of 5 MPa, 10 MPa and 15 MPa for single and mixed grain sizes, respectively. The stress–strain curves are shown in Figs. 4, 5 and 6.

As can be seen in Fig. 4, at 5 MPa axial pressure, the strain was 23.8% for 0–5 mm grain size, 25.5% for 5–10 mm grain size, 28.9% for 10–15 mm grain size, 24.7% for 15–20 mm grain size, and 20.8% for mixed grain size. Meanwhile, under this axial pressure, the strain of the three particle sizes, 0–5 mm, 5–10 mm, and 10–15 mm, increases with the increase of the particle size. Generally, as the particle size increases, the pore space increases in the same volume, making the compression increase, but the test results in the two groups of 10–15 mm particle size and mixed particle size instead decreased. The reason may be analysed because of the greater strength of the crushed gangue particles in the airspace, and at the same time the level of axial pressure is smaller, resulting in the stopping of axial loading before the smaller gangue that has been crushed by the secondary crushing has been able to fill the pore space completely again. That is, the gangue under the horizontal axis pressure failed to be in a fully compacted state, so there are 10–15 mm particle size and mixed particle size of the two groups of strain but decreased in the situation.

As can be seen in Fig. 5, at 10 MPa axial pressure, the strain was 25.7% for 0–5 mm grain size, 32.1% for 5–10 mm grain size, 37.1% for 10–15 mm grain size, 36.3% for 15–20 mm grain size, and 25.2% for mixed grain size. As with the 5 MPa axial pressure, at this axial pressure, the amount of strain for the three grain sizes, 0–5 mm, 5–10 mm, and 10–15 mm, increases with increasing grain size. Still in the 10–15 mm particle size and mixed particle size of the two groups of test results do not rise rather than fall, the reason may still be due to the crushed gangue particles in the airspace gangue strength is greater, at the same time, the level of axial pressure is smaller. The gangue under the pressure of the horizontal axis is not in a fully compacted state, so there are 10–15 mm particle size and mixed particle size of the two groups of strain variables appear not to rise but to fall phenomenon.

As can be seen in Fig. 6, at 15 MPa axial pressure, the strain was 32.3% for 0–5 mm grain size, 42.4% for 5–10 mm grain size, 46.0% for 10–15 mm grain size, 48.0% for 15–20 mm grain size, and 37.3% for mixed grain size. Different from the test results at 5 MPa and 10 MPa axial pressure, the strain increases with increasing grain size when the axial pressure reaches a certain level. By analysing the graphs of the compression test results at different axial pressures, the following patterns were obtained:

1. Different particle size gangue in the axial pressure, the stress–strain curve is different, but are a nonlinear curve, and its trend is the same, like an exponential function distribution.
2. When the axial pressure reaches a certain threshold, with the increase of particle size, the deformation shows an increasing trend.
3. Under the action of axial load, the deformation of crushed gangue in the mining area mainly experiences the following three stages:

The first stage is the initial compaction stage. At this stage, the broken gangue is in a natural accumulation state, and there are a large number of pores between the particles. When subjected to load, the relative sliding between the particles occurs, and the pores decrease rapidly. At this time, the σ-ε curve shows that under a smaller load, a larger deformation will occur.

The second stage is the stable deformation stage, after the first stage of compression, the pore space is reduced in large quantities, in order to make the gangue further deformation, the need for a larger axial load, with the increase in pressure, the pore space is constantly filled, at the same time, this stage there will be a second gangue crushing filling phenomenon, that is, the gangue is crushed into a smaller size of the grain size of the gangue to fill the pore space further, but in general, with the increase in stress, the rate of deformation of the strain variable has been significantly reduced, so that *σ-ε* curves show a clear upward concave tendency, the stress strain curve show the change rule of the form of an exponential.

In the third stage, the crushed gangue enters the elastic–plastic deformation stage. After the compression of the first two stages, the crushed gangue is gradually compacted and its structure becomes more compact and stable. At this point, the crushed gangue has been transformed into a continuous medium, similar to a complete rock, this stage requires the application of large axial pressure to produce deformation. The *σ-ε* curves show a sharp increase in stress with increasing strain in a linear manner, exhibiting a clear linear characteristic.

#### Analysis of lateral pressure curve of coal gangue with different particle size

**Figure 7:**
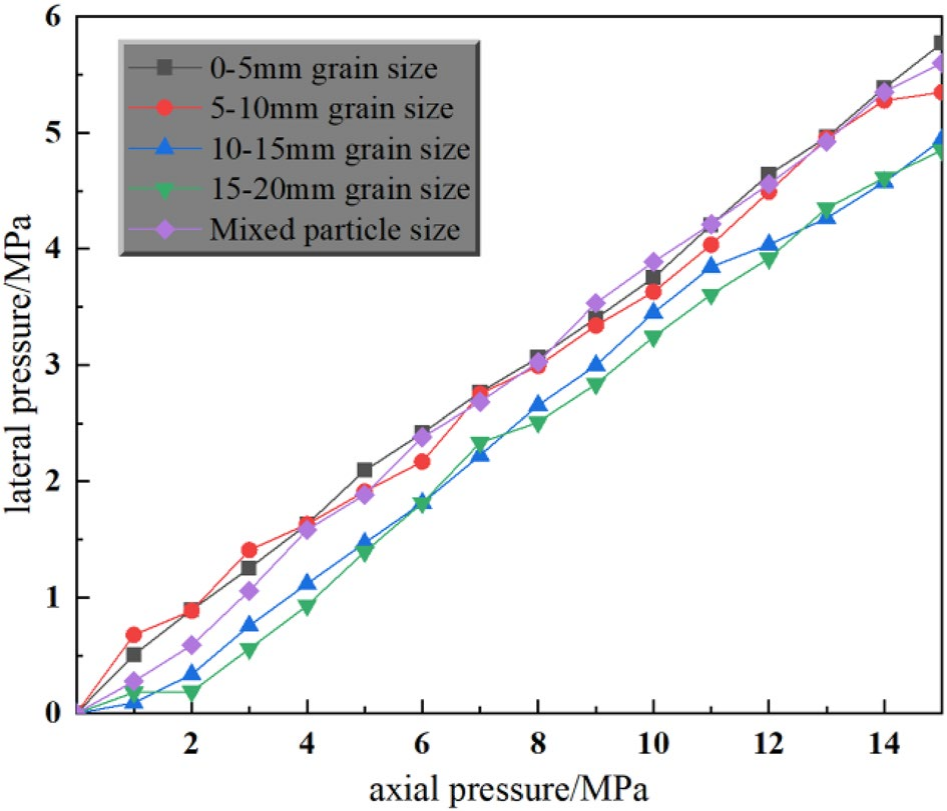
Relationship between axial pressure and lateral pressure of crushed gangue under different grain sizes.

The crushed gangue will be displaced laterally in the process of being compacted, and the wall of the cylinder will be subjected to a certain amount of lateral pressure due to the presence of the steel cylinder, which restricts the displacement of the gangue. From the above gangue compression stress–strain curve analysis, it can be seen that when the level of axial pressure is low, it can't reflect the evolution law of different grain sizes in compression very completely. Therefore, the study analyzes the lateral pressure curve under 15 MPa axial pressure, as shown in Fig. 7, and the fitting function is shown in Table 2..

**Table 2:** Fitting function table of lateral pressure curve of gangue with different particle size.

Particle size	Function	R^2^
0–5 mm	$y=0.38422x$	0.99929
5–10 mm	$y=0.37622x$	0.99842
10–15 mm	$y=0.32923x$	0.99682
15–20 mm	$y=0.32143x$	0.99467
Mixed particle size	$y=0.38086x$	0.99944

From Fig. 7, it can be obtained that in the process of side-limit compression test, the lateral pressure produced by gangue specimens of different grain sizes on the restraining wall is directly proportional to the axial pressure it is subjected to, which can be expressed by a linear function y = a*x, where a is the lateral pressure coefficient. By analysing the above lateral pressure results, the fitting function of lateral pressure for gangue with different grain sizes is.

In the process of coal gangue filling of the mining area, the compaction of the overlying rock layer will lead to lateral expansion of gangue particles, which in turn will generate lateral pressure on the surrounding gangue retaining wall and the filling body along the hollow retaining lane. According to relevant research conclusions, the size of lateral pressure can be deduced through the lateral pressure coefficient, which can provide a scientific basis for the strength design of the retaining gangway wall and the filling body along the air retaining lane to ensure its stability, so as to achieve a better filling effect.

## Model generation and fine-scale parameter calibration

### Generation of gangue model

**Figure 8:**
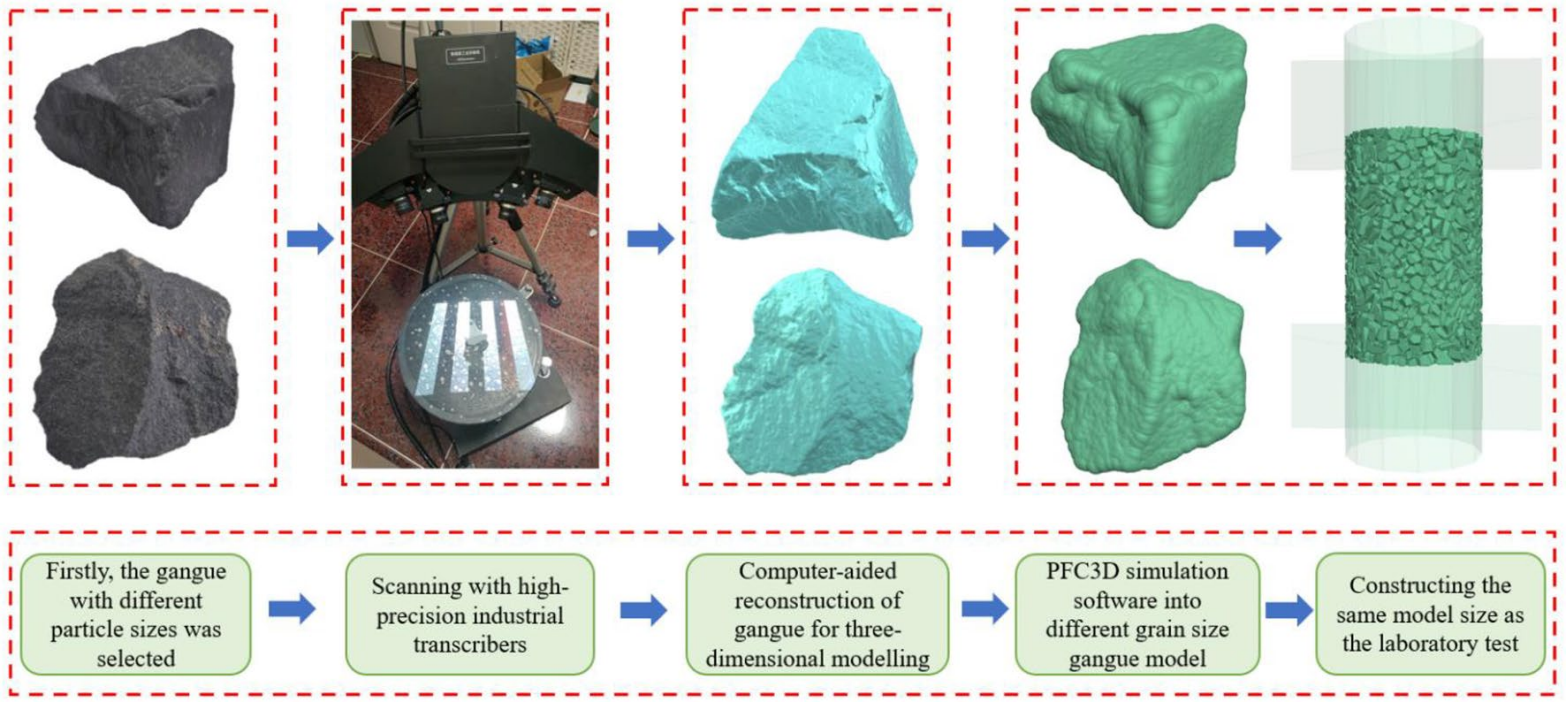
Generation process of different gangue particles.

Firstly, different particle size gangue is selected, and the outer surface of gangue is sprayed with colouring penetrating flaw detector, and put under high precision industrial scanning machine to scan and record with multi-angle scanning. And the scanned gangue was imported into FlexScan3D software, and computer-aided design was used to establish the outer contour model of gangue with different grain sizes in three dimensions, and the three-dimensional model (stl) file of gangue was exported. The files were imported into the particle flow simulation software PFC3D and gangue model specimens with different particle sizes and shapes were generated. The model is constructed as follows: Firstly, a steel cylinder of the same dimensions as in the laboratory tests was constructed (100 mm diameter), as well as a fixed gangue filling height (150 mm high). Next, the gangue particle model generated in the PFC 3D software is filled in the model according to the needs of the test (gangue of different particle sizes) and a rigid gangue filling body is composed, as shown in Fig. 8.

### Calibration of gangue fine view parameters

**Figure 9:**
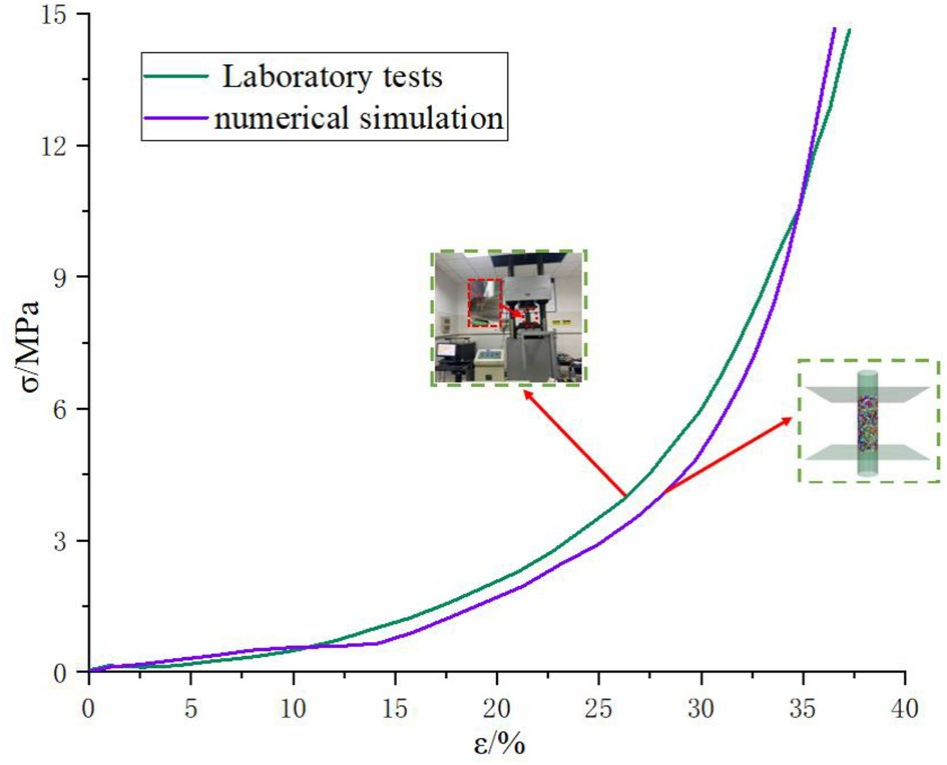
(0–20 mm) gangue particle physical experiment and numerical simulation results.

The Clump model in PFC3D numerical simulation software was used to simulate gangue particles with different grain sizes. Since gangue particles are considered as rigid bodies, it is not possible to specify gangue physical parameters directly. The macroscopic mechanical behaviour of gangue depends on the fine-scale behaviour of inter-particle contact, porosity, force chains, and displacement. The objects studied in this paper are gangue particles of different grain sizes, whose microscopic parameters are difficult to obtain directly. Therefore, taking (0–20 mm) mixed gangue as an example (the proportion of different stages of mixed particle size is the same as the proportion of gangue in the laboratory test), the micro-parameters are calibrated through a large number of numerical simulations, repeated tests, and through the trial-and-error method. Through the laboratory (0–20 mm) mixed gangue compression test during the stress–strain curve and numerical simulation of the compression test stress–strain curve, a number of iterative debugging of the numerical model micro-parameters, until the numerical simulation of the stress–strain curve and the laboratory experimental results of the basic fit. There is a better correspondence for the stress–strain after commissioning, as shown in Fig. 9. In order to better match the parameters of the physical experiments, the numerical simulation microscopic parameters are shown in Table 3.

**Table 3:** Calibration of the main microscopic parameters of gangue particles.

Particle–particle contact modulus (MPa)	Particle–wall contact modulus (MPa)	Particle friction coefficient	Particle density (g/cm^3^)
70	700	0.5	2.67

The gangue model was successfully constructed through logarithmic simulation, which can accurately obtain the mechanical properties of the specimen with a high degree of reduction. The results of the various data show that there is some error between the numerical model test results and the physical experiments, but this deviation is not very large.

## Numerical simulation analysis

### Natural accumulation porosity

**Figure 10:**
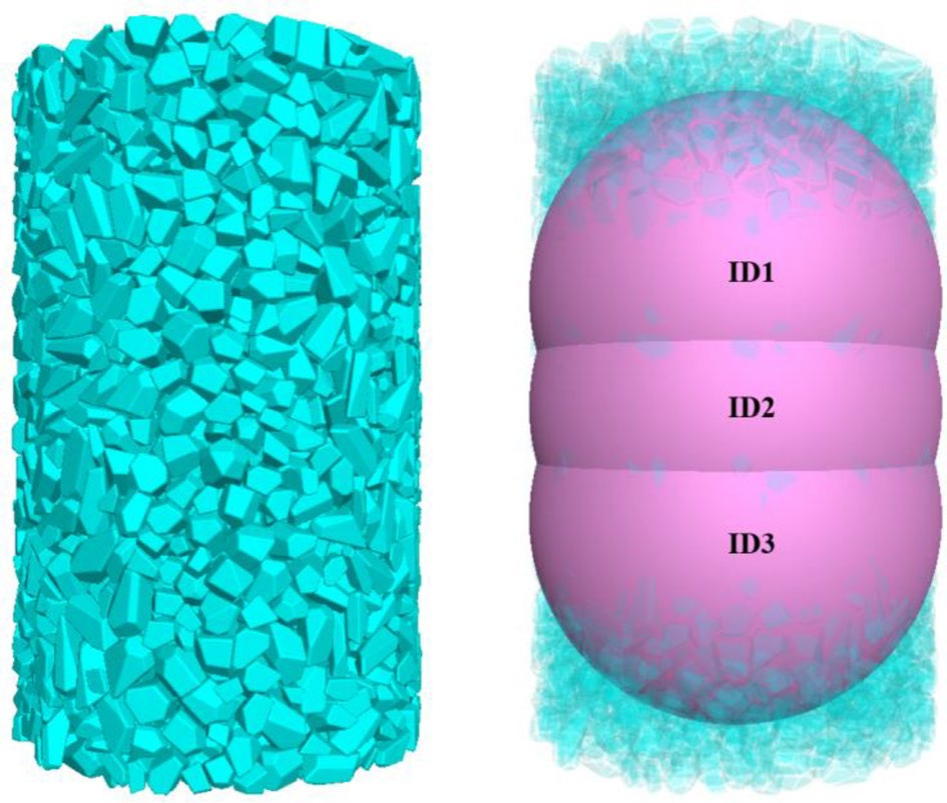
Natural accumulation of gangue, measuring balls.

Pore space is one of the important factors leading to the initial deformation of gangue filling, and its formation is mainly affected by the arrangement, shape and particle size distribution of gangue particles inside the filling body of the hollow area. These factors together determine the porosity of gangue-filled materials, which in turn affects their filling stability and mechanical properties. In order to investigate the relationship between different grain sizes and porosity, the model will be constructed based on different grain size gangue materials used in laboratory tests. Through the numerical simulation method, the porosity of the natural accumulation of coal gangue with different grain sizes will be measured, and in-depth discussions will be conducted to analyse the mechanical behaviour of the gangue affecting the filling of coal gangue in the mining area. In order to study the change of porosity during natural accumulation of gangue, three measuring balls are set in the numerical model, and the distribution of measuring balls is shown in Fig. 10.

**Figure 11:**
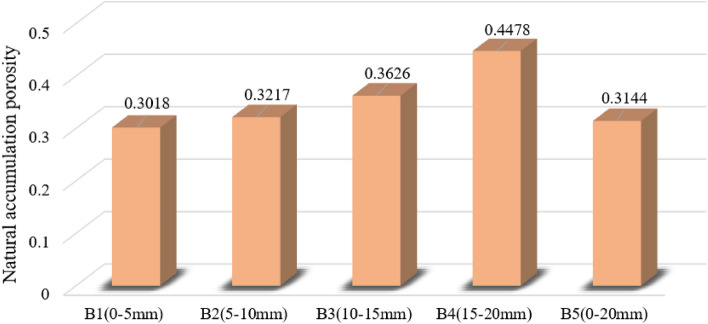
Porosity of gangue with different grain sizes in natural accumulation.

After the numerical simulation of the gangue specimen filling body reached a completely stable state, the porosity of the three measuring sphere regions during the natural accumulation process was recorded in detail. The average of the porosities measured by these three measuring spheres was taken to give the final void ratio. The experimental results are shown in Fig. 11, the natural accumulation void ratio simulated by different grain size gangue specimens.

(1) For the gangue specimens (B1–B5), the natural accumulation porosity was ranked as B1 < B5 < B2 < B3 < B4, with 0.3018, 0.3144, 0.3217, 0.3626 and 0.4478, respectively. In the numerical model, there is a certain relationship between the natural stacking porosity of gangue particles and particle size, and the natural stacking porosity of gangue particles will show a rising trend with the increase of particle size. This phenomenon is due to the gangue particle size increases, its length, width and height will also gradually become larger, the more irregular gangue shape, the more likely to contact each other between the gangue particles to form a shelf structure. This will lead to the gangue in the hollow area filling degree is low, gangue and gangue contact between the gangue is not close, the gangue between the pore will be larger, less pressure resistance. B1 (0–5 mm) shape of the gangue particles are more regular and flat, length, width and height are more uniform, the gangue is arranged compactly, so the porosity of gangue specimen B1 is relatively low, high degree of filling.

(2) For specimens B2 (5–10 mm) and B3 (10–15 mm), with the increase of gangue particle size, its natural accumulation porosity shows a gradual increase trend, which is due to the increase of gangue particle size, the larger the volume occupied by the particles. The more the gangue is haphazardly filled in the test, the greater the properties exhibited, and therefore the natural accumulation porosity variation increases accordingly. For the large-size gangue specimen B4 (15–20 mm), the natural stacking porosity was higher than that of gangue of all combined sizes. This is due to the large size of the gangue particles is difficult to achieve close contact with each other, and the gangue block is easy to form a shelf structure between, thus resulting in B4 gangue specimen porosity will be higher.

(3) For B5 (0–20 mm) gangue particles in the natural accumulation of the process, the porosity is second only to B1, this is due to the small particle size gangue to fill in the large particle size gangue overhead pore, gangue particles between the contact tends to be close to the pore to be filled, the gangue between the different particle size of the gangue for the adjustment and optimisation of each other, so as to make the B5 specimen of the porosity of the low, high degree of filling.

The above results show that according to the different particle sizes of gangue materials used in the simulation test, different particle sizes of gangue combination specimens were obtained to have a greater impact on the natural accumulation porosity. The larger the grain size, the more significant the gangue profile, resulting in a more irregular gangue block shape, which in turn results in a higher natural stacking porosity of the specimen. When a mixture of gangue with multiple particle sizes is used, the natural accumulation porosity of the specimen can be significantly reduced. However, this law only applies in a certain range of particle size, with the gangue particle size reduction or increase, this law will change accordingly.

### Overall deformation of appearance

**Figure 12:**
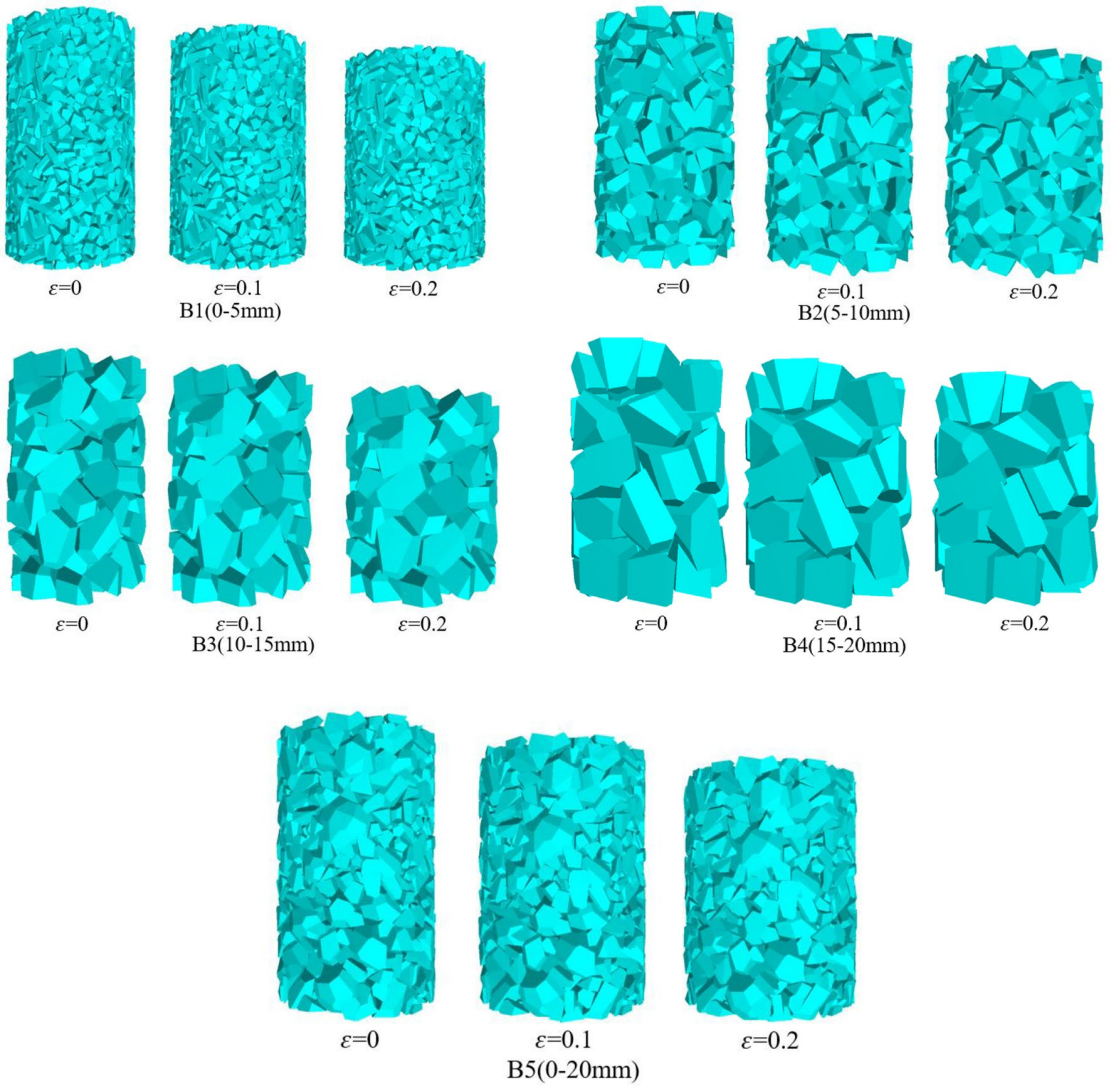
Stacking change of gangue with different particle size in compression process.

Simulating the change of gangue with different grain sizes under different filling depths is transformed into the change of microscopic movement and mechanical properties under different circumferential pressure conditions based on the analysis of the gangue pressure measurement curves mentioned above. Due to the limitation of space, this paper mainly analyses the macroscopic changes and microscopic evolution of gangue with different grain sizes when the enclosing pressure is 2 MPa. Under the condition of *σ* = 2 MPa, the overall appearance deformation of gangue specimens (B1-B5) with different grain sizes, when *ε* = 0, 0.1 and 0.2 during compression, is shown in Fig. 12.

The following conclusions are drawn from Fig. 12:

1. With the increasing axial stress, the gangue of different grain sizes began to be gradually compacted, resulting in the lateral deformation of the specimen showed a trend of gradual expansion around. Gangue loading at the initial stage, under the constraint of the surrounding pressure, the gangue and gangue extrusion deformation occurs with each other, and from the overall appearance of the specimen to see the densification is better, this is due to the initial state of the gangue is more loose, the gangue between the pore size is larger, with the compression process continues, the gangue is gradually compacted.
2. In the process of compression, the lateral displacement of the gangue specimen is constrained, the gangue specimen space is gradually compressed, and the gangue begins to squeeze each other. Because in the simulation, gangue particles are regarded as rigid body, particles can not be compressed to destroy, gangue each other began to mosaic, small size gangue between the performance is not obvious, large size gangue some have been extruded bite together.

### Changes in particle displacement

**Figure 13:**
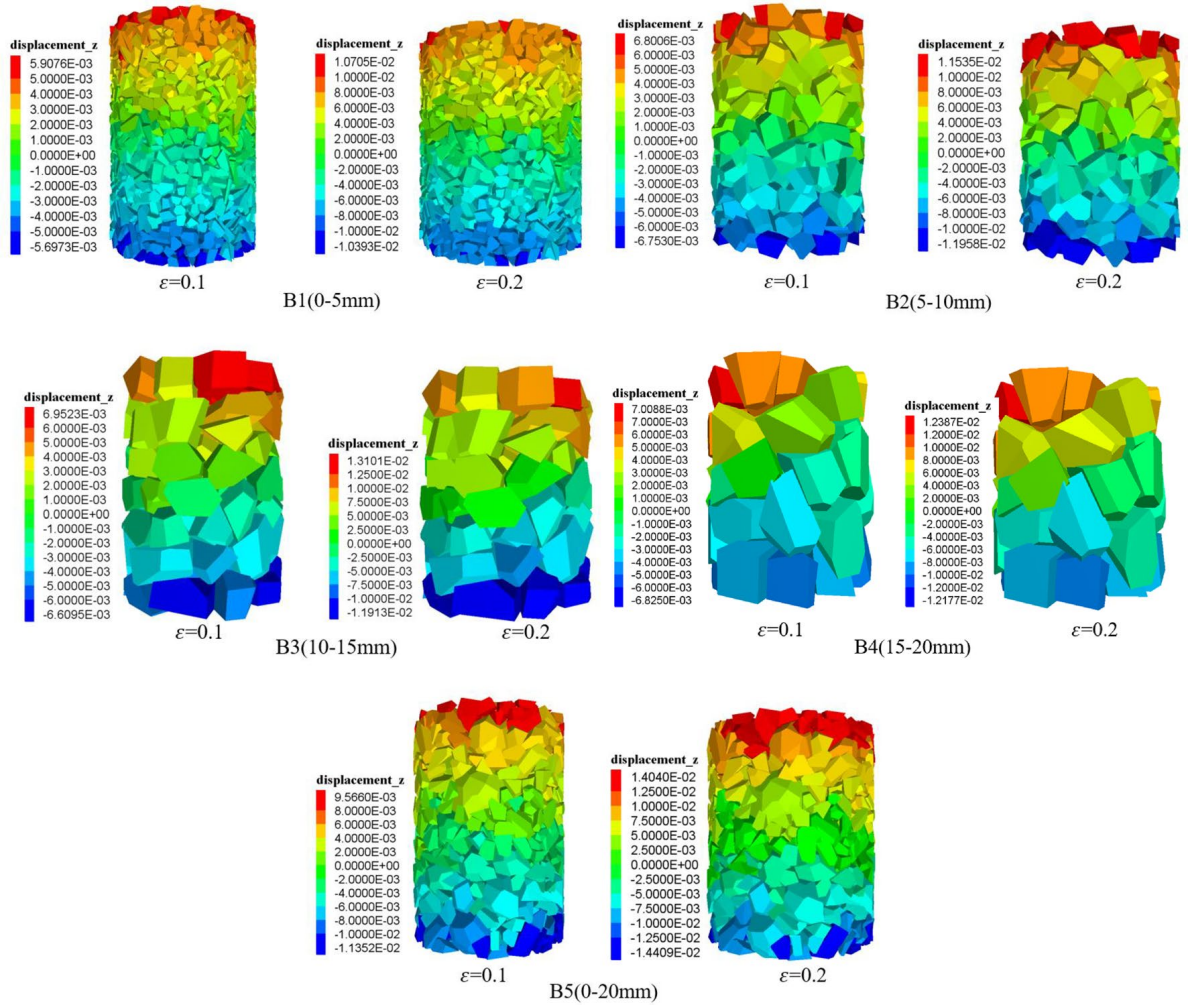
Cloud diagram of axial displacement of gangue particles under different strains (*σ* = 2.0 MPa, *ε* = 0.1, *ε* = 0.2).

**Figure 14:**
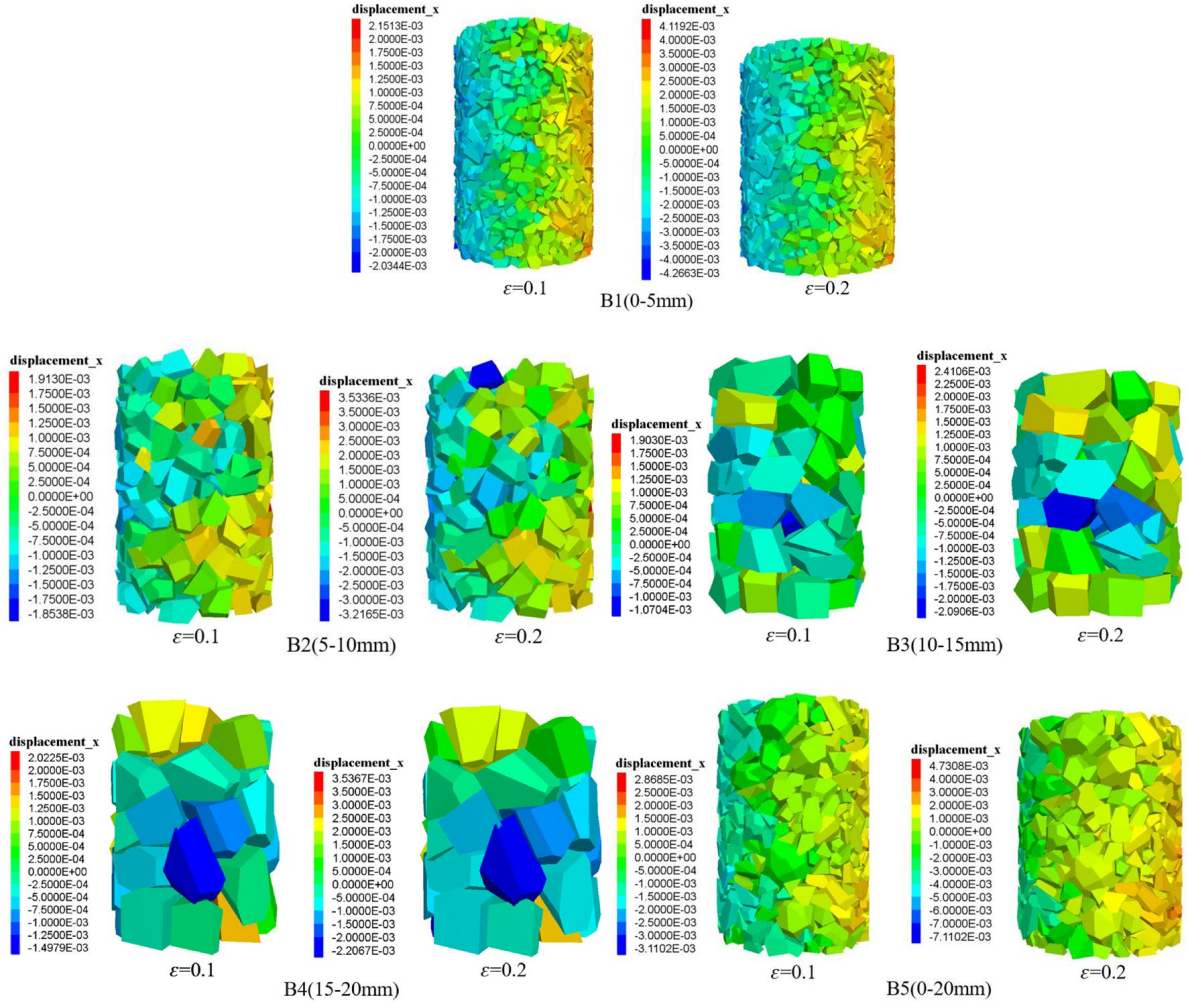
Lateral displacement cloud of gangue particles under different strains (*σ* = 2.0 MPa, *ε* = 0.1,* ε* = 0.2).

Under the 2 MPa circumferential pressure condition, when the strains of gangue particles are *ε* = 0.1 and *ε* = 0.2, the axial displacement cloud and lateral displacement cloud of gangue particles with different grain sizes are shown in Figs. 13 and 14, respectively.

(1) Under the condition of 2 MPa surrounding pressure, the displacement of gangue particles under different strain states has a similar law. The amount of change in axial displacement of gangue particles shows a decrease from top to bottom. Gangue with the same laminar distribution, i.e. different axial displacement of gangue particles at the same filling height. The displacement of the gangue specimen is maximum at the top and decreases gradually towards the bottom. The displacement of gangue particles shows a more symmetrical distribution, there are obvious axial displacement changes at the top of the specimen, and specimen compression is the main factor of axial displacement of gangue particles.

(2) In the process of compression deformation, it is observed that the gangue particles in the axial displacement of the top of the obvious triangular area, B1, B2, B5 appeared more obvious, and the gangue particles in the triangular area of the axial displacement is significantly higher than in the same layer of the other gangue particles. This is due to the B1, B2 gangue particle size is small, B5 in the small particle size gangue to fill the large particles of gangue overhead space. The absence of distinct triangular regions in B3 and B4 may be caused by the large gangue grain size or because of the size limitation of the specimen modelling space. An inert triangle area was observed at the bottom of the model. In this area, the axial displacement of the gangue particles was lower than that of other particles in the same layer.

(3) Different sizes of gangue particles have different degrees of influence on the change of displacement during compression. When the model is compressed to *ε* = 0.2, for the large-size gangue specimen, the axial displacement of gangue particles at the top of the specimen shows the distribution of individual particle changes, and the delta is not obvious, which is due to the larger top gangue particles in the specimen and the number of fewer gangue particles. For B1–B4 gangue particles of different particle size specimens, the top displacement of the triangle with the increase in particle size gradually becomes smaller, while B5 mixed particle size gangue has a similar change with B1, due to the small particle size gangue filled in the pore space of the large particle size gangue, the particles in contact with the pore space becomes more tightly and the pore space is fully filled. The axial displacement of the particles in the B1-B5 gangue specimens has some differences, and the maximum axial displacement is 0.01 m, 0.011 m, 0.013 m, 0.012 m, 0.014 m. According to simulation results, it is concluded that as the gangue size increases, the gangue particles are more and more irregular, and when subjected to the compression effect, the irregularly shaped gangue particles show a different axial displacements.

(1) On one side of the specimen, triangular areas of lateral movement of gangue particles appear in B1, B2, and B5. The lateral displacement on the outside of the gangue specimen is greater than the lateral displacement at the centre, which is the main cause of specimen expansion. The displacement of gangue particles under different strains is different, small-size gangue particles move more actively, and large-size gangue particles are not significantly displaced. Gangue particles in a certain compression, in order to overcome the gangue between the mutual occlusion and friction so as to move, so that the specimen inside the gangue particles in the lateral displacement of the main reason.

(2) In the compression process, gangue specimens with different grain sizes showed different transverse motion displacements. When the gangue specimen is compressed to* ε* = 0.2, there are obvious triangular zones in B1 and B5, and there are no obvious triangular zones in the transverse displacement of gangue particles for the gangue with large particle sizes of B2–B4. B5 gangue specimen lateral displacement changes similar to B1, this is due to the small grain size gangue to fill the large grain size gangue overhead space. B1–B4 gangue specimens, the amount of change in transverse displacement gradually becomes smaller as the particle size becomes larger. The maximum transverse displacements of gangue specimens with different grain sizes were ranked as B5 > B1 > B4 > B2 > B3, which were 0.0047 m, 0.0041 m, 0.0035 m, 0.0035 m and 0.0024 m, respectively. According to the simulation results, when the particle size between the gangue of the filling body is smaller, the pore space of the gangue also decreases, and the lateral movement of gangue particles is more and more significant in the compression process.

According to the conclusion of the above simulation study, in the coal gangue filling, the particle size of the filling gangue should be moderate, small particle size gangue crushing cost is high, large particle size gangue is easy to rack between the larger pores, compression and deformation of the amount of compression, the stability is poor. Mixed grain size gangue reduces crushing cost and fills the overhead area between large grain size gangue, making the gangue in close contact with each other, with better pressure bearing and controlling deformation. And to properly strengthen the tamping filling work surface, so that the gangue on both sides of the gangue has a high degree of compactness and bearing capacity, to achieve the gangue in the same position in the overlying rock layer pressure together under the action of the sinking, and to the side of the lane filling body and gangue retaining walls for reinforcement, in order to enhance the gangue of the anti-pressure.

### Skeleton force chain evolution

**Figure 15:**
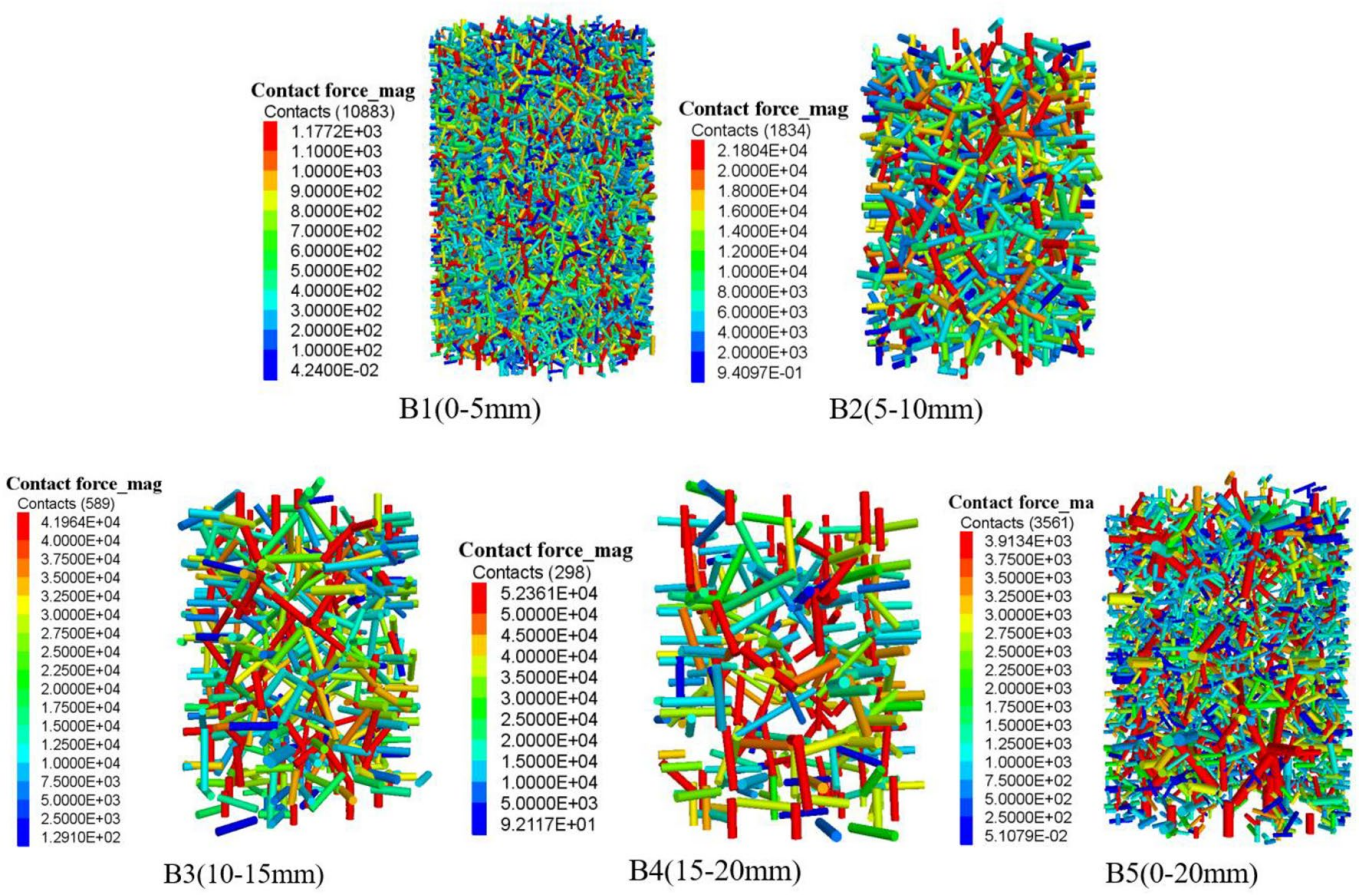
Distribution of force chain of coal gangue samples with different particle sizes (*σ* = 2.0 MPa, *ε* = 0.2).

**Figure 16:**
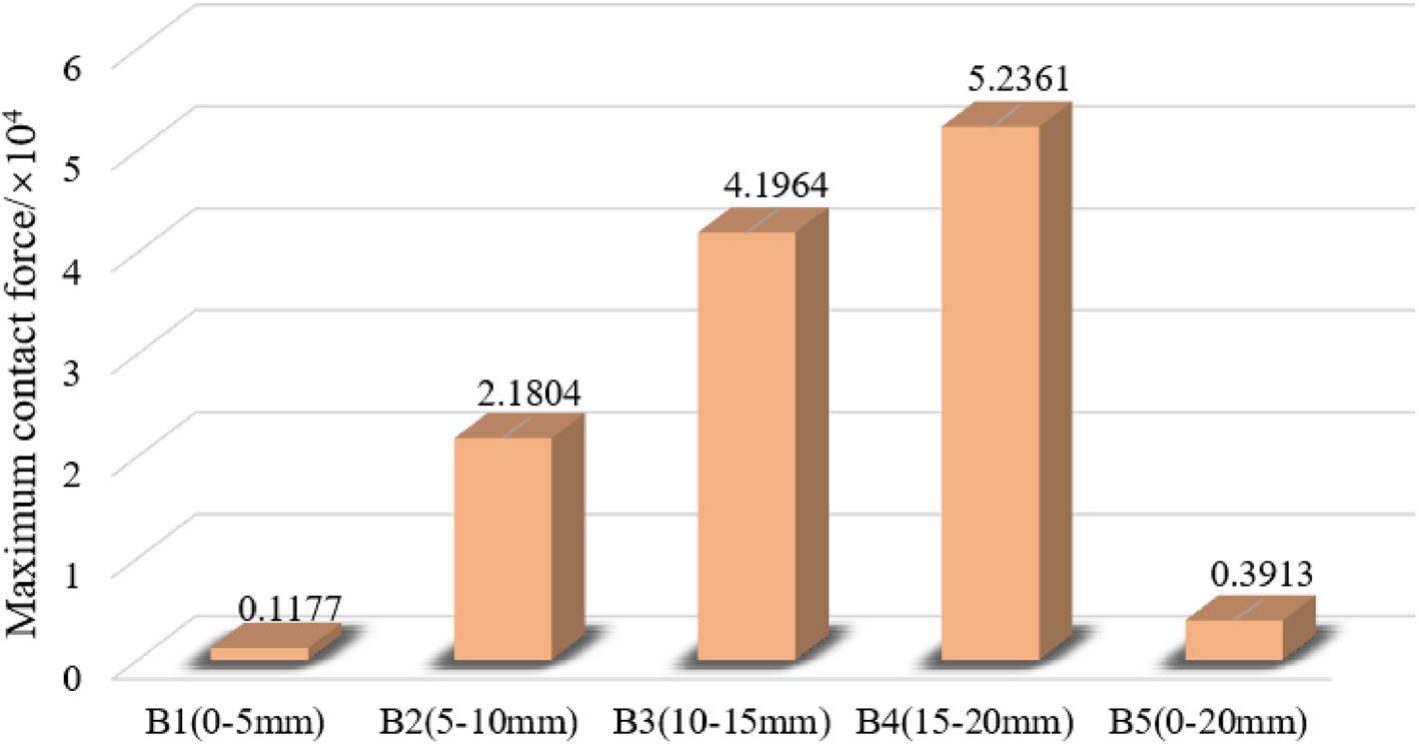
Maximum contact force of gangue specimens with different grain sizes (σ = 2.0 MPa, ε = 0.2).

**Figure 17:**
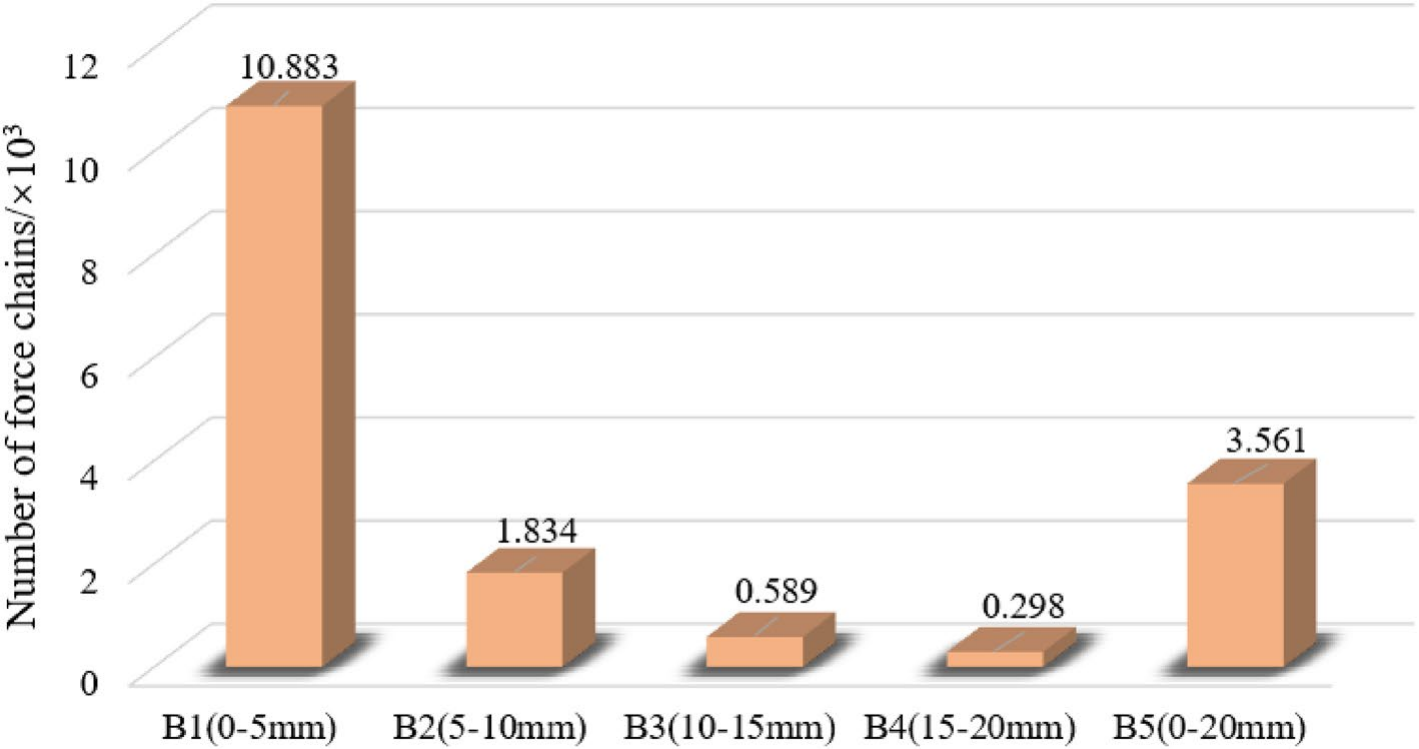
Force chain numbers of gangue specimens with different grain sizes (σ = 2.0 MPa, ε = 0.2).

Different particle size gangue under the condition of *σ* = 2.0 MPa, when *ε* = 0.2, the gangue specimen internal particle contact force chain distribution is shown in Fig. 15, gangue particles specimen internal contact force chain total number is shown in Fig. 16, the maximum contact force is shown in Fig. 17. In the gangue particles compression process, the skeleton force chain is composed of neighbouring gangue particles between the transfer of force composed of force chain structure, mainly to withstand most of the axial load compression force, and surrounded by the wall constraints, and to ensure its structural stability. The more red force chains in the diagram, the higher the force chain contact force and the higher the stability.

1. from the figure can be obtained, the same peripheral pressure of different particle size gangue specimen compression to *ε* = 0.2, with the compression of the specimen, the specimen inside the skeleton chain of force between the mutual contact of the gradual enhancement of the total number of skeleton chain of force and the gangue particles of the maximum contact force between the gradual increase, indicating that the larger the axial pressure, the same strain state of the gangue the larger the load bearing capacity.
2. During the simulation, gangue specimens of different grain sizes were gradually compacted. With the gangue particles in close contact with each other, and the formation of a stable skeleton structure. The skeleton force chain is completely penetrated throughout the sample. In the compression process, the gangue of different particle sizes constantly occurs the instability and reconstruction of the skeleton force chain structure, which makes the number of force chains in the specimen gradually increase, thus enhancing the stability of the gangue.
3. There are differences in the stability of the skeleton force chain structure in gangue particles of different grain sizes. When the strain value is 0.2, the stability of the skeleton force chain in gangue specimens of each grain size is ranked as follows: B1 > B5 > B2 > B3 > B4. This is because the gangue particles of different grain sizes will produce different sizes of pores, which form spaces of different sizes when racking, thus affecting the stability of the skeleton force chain structure. Specifically, gangue particles with large particle size are more likely to lead to instability and failure of the force chain. Therefore, when selecting and using gangue materials, the influence of its particle size on the structural stability of the skeleton force chain should be fully considered. When *ε* = 0.2, the maximum contact force of gangue particles of different sizes is: B4 > B3 > B2 > B5 > B1, it can be seen that the same circumferential pressure conditions of different sizes of gangue bearing performance has obvious differences, the maximum contact force of large size gangue is obviously higher than that of small size gangue particles.
4. Among these skeletons, those with a particle size range of B5 are the most pronounced and form a solid structure, which is due to the filling of pores with small gangue sizes and the dense contact between gangue particles. The force chain structure of the specimen gradually formed a relatively stable triangular form, the appearance of this form effectively improves the specimen's resistance to deformation of the compression, deformation resistance, making it more stable and reliable. The gangue particles of particle size B1 reach a relatively stable stage after a rapid compaction stage, the gangue particles of B2, B3 and B4 have relatively fewer force chains, the gangue particle size is larger, and there is still a sliding process between the gangue particles.

## Conclusion

This paper comprehensively analyses the mutual contact, extrusion, deformation and displacement in the gangue compression process through the gangue compression change characteristics, and draws the following key conclusions from the macroscopic change and microscopic evolution perspectives:

1. The compression test results of gangue with different particle size under 5 MPa, 10 MPa and 15 MPa axial pressure show that: when the axial pressure reaches a certain threshold, the strain increases with the increase of particle size. Different particle size gangue in the axial pressure, the stress–strain curve is different, but are a nonlinear curve, and its trend of change are the same, like an exponential function distribution.
2. Different particle size gangue in 15 MPa axial pressure cylinder side pressure results show that: in the compression process, the cylinder wall side pressure and its bearing axial pressure shows a positive relationship; when the particle size is larger, the gangue of large particle size will occur in the pressure of the secondary crushing, resulting in a short-lived release of stress, and the side pressure curve shows jumping fluctuations in the loading of the early stage of the phenomenon is very significant, along with the axial pressure is constantly growing, the pore space between the crushing of gangue gradually filled, so the fluctuation amplitude of the later stage of the slowly reduced.
3. The gangue specimens with different particle sizes have a significant effect on the natural stacking porosity. With the increase of gangue particles, the natural accumulation porosity showed an increasing trend. When the gangue with mixed particle size was used, the natural accumulation porosity decreased significantly. In addition, as the gangue particle size decreases, the axial and transverse displacement phenomenon of gangue particles becomes more and more significant, which improves the stability of the skeleton force chain structure.
4. During the compression process, the stability of the force chain in the gangue specimens of each particle size is ranked as follows: B1 > B5 > B2 > B3 > B4. The large-size gangue particles are more prone to instability and failure of the force chain. According to the different gangue particle size, the maximum contact force of the specimen is ranked as follows: B4 > B3 > B2 > B5 > B1, it can be seen that the same peripheral pressure conditions of different particle size gangue bearing performance has obvious differences, the maximum contact force of large size gangue is obviously higher than the small size gangue particles.

## Data Availability

The data used and analysed during the research for this paper can be obtained from the corresponding author upon reasonable request.
